# SEOM-GEICAM-SOLTI clinical guidelines for early-stage breast cancer (2022)

**DOI:** 10.1007/s12094-023-03215-4

**Published:** 2023-06-16

**Authors:** Francisco Ayala de la Peña, Silvia Antolín Novoa, Joaquín Gavilá Gregori, Lucía González Cortijo, Fernando Henao Carrasco, María Teresa Martínez Martínez, Cristina Morales Estévez, Agostina Stradella, María Jesús Vidal Losada, Eva Ciruelos

**Affiliations:** 1 Department of Medical Oncology, Hospital G. Universitario Morales Meseguer, University of Murcia, Av. Marqués de los Vélez, s/n, 30008 Murcia, Spain; 2 Department of Medical Oncology, Complexo Hospitalario Universitario, A Coruña (CHUAC), Coruña, Spain; 3 Fundación Instituto Valenciano de Oncología (IVO), Valencia, Spain; 4 Medical Oncology Department, Hospital Universitario Quirónsalud, Madrid, Spain; 5 Hospital Universitario Virgen Macarena, Seville, Spain; 6 Medical Oncology Department, INCLIVA Biomedical Research Institute, Hospital Clínico of Valencia, University of Valencia, 46010 Valencia, Spain; 7 Hospital Universitario Reina Sofía, Córdoba, Spain; 8 Medical Oncology Department, Institut Català d’Oncologia. L’Hospitalet,, Barcelona, Spain; 9 Hospital Clínic, Barcelona, Spain; 10 Medical Oncology Department, Breast Cancer Unit, University Hospital 12 de Octubre, Madrid, Spain and HM Hospitales, Madrid, Spain

**Keywords:** Early breast cancer, Adjuvant, Neoadjuvant, Follow-up

## Abstract

Breast cancer is the leading cause of cancer in women in Spain and its annual incidence is rapidly increasing. Thanks to the screening programs in place, nearly 90% of breast cancer cases are detected in early and potentially curable stages, despite the COVID-19 pandemic possibly having impacted these numbers (not yet quantified). In recent years, locoregional and systemic therapies are increasingly being directed by new diagnostic tools that have improved the balance between toxicity and clinical benefit. New therapeutic strategies, such as immunotherapy, targeted drugs, and antibody–drug conjugates have also improved outcomes in some patient subgroups. This clinical practice guideline is based on a systematic review of relevant studies and on the consensus of experts from GEICAM, SOLTI, and SEOM.

## Introduction

Breast cancer is a major public health problem given its high incidence, prevalence, and mortality, representing the most common cancer among women in Spain and accounting for 35,000 new cases per year. Moreover, it is the leading cause of cancer-related mortality in the female population, responsible for 6651 cancer deaths per year [1].

Breast cancer is a heterogeneous disease with marked clinical and biological heterogeneity, leading to many therapeutic decisions being individualized depending on molecular and clinical characteristics. Despite the success in implementing early breast cancer detection campaigns, up to one in three patients will develop metastases. Moreover, approximately one in 8–9 new diagnoses currently present as metastatic disease; these figures may be worse because of screening delays across the country due to COVID19 pandemic [2].

The aim of these guidelines was to summarize and categorize current evidence that arises useful clinical therapeutic recommendations in the clinical practice.

## Methodology

This guideline is based on a systematic review of relevant published studies and with the consensus of ten oncologists who are experts in treatment of breast cancer from GEICAM, SOLTI, and SEOM, as well as an external review panel comprising two experts designated by SEOM. The Infectious Diseases Society of America-US Public Health Service Grading System for Ranking Recommendations in Clinical Guidelines Infectious Diseases Society of America-US Public Health Service Grading System for Ranking Recommendations in Clinical Guidelines [3] has been used to assign levels of evidence and grades of recommendation (Table 1).

**Table 1:** Strength of recommendation and quality of evidence score

Category, grade	Definition
Strength of recommendation	
A	Good evidence to support a recommendation for use
B	Moderate evidence to support a recommendation for use
C	Poor evidence to support a recommendation
D	Moderate evidence to support a recommendation against use
E	Good evidence to support a recommendation against use
Quality of evidence	
I	Evidence from ≥ 1 properly randomized, controlled trial
II	Evidence from ≥ 1 well-designed clinical trial, without randomization; from cohort or case-controlled analytic studies (preferably from > 1 center); from multiple time series; or from dramatic results from uncontrolled experiments
III	Evidence from opinions of respected authorities, based on clinical experience, descriptive studies, or reports of expert committees

## Diagnosis and staging

To diagnose breast cancer, a clinical, radiological, and pathological examination is necessary. Staging should be conducted according to TNM and the AJCC system [4]. A proper anamnesis with personal and family history and a complete physical examination (palpation of the breasts, regional lymph nodes, and assessment for distant metastases) should be performed. Furthermore, the following radiological tests should be performed to establish an accurate diagnosis:

1. Bilateral mammography and ultrasound of the breast and regional lymph nodes (I, A) [5]. New techniques, such as 3D mammography or 3D ultrasound, increase diagnostic accuracy, but are not routinely implemented.
2. Core needle biopsy (preferably under ultrasound or stereotactic guidance) (I, A).
3. Fine needle aspiration or core biopsy of suspicious lymph nodes is recommended (II, A).
4. Breast magnetic resonance imaging (MRI) is optional (I, B) and should be only considered in cases of positive axillary nodes; occult primary breast cancer; Paget’s disease of the nipple; lobular carcinoma; multifocal, multicentric lesions, and breast cancer implants. It is also recommended pre- and post-neoadjuvant treatment to define the extent of disease and monitor treatment response (III, A) [6].
5. Additional studies: Evaluation of cardiac function is imperative when using anthracyclines or antiHER2-targeted therapies (I, A) [7]. Laboratory tests (complete blood count, liver and renal function, alkaline phosphatase, and calcium) are routinely performed, but do not appear to improve detection of occult metastatic disease (III, C) [8]. Additional systemic staging should be contemplated when disease is detected in stage III or when signs, symptoms, or laboratory values indicate possible metastasis. This more comprehensive study includes chest, abdominal, and pelvic imaging, and bone scan (III, B). PET/CT may be of use when traditional imaging test are inconclusive (III,A) or in cases of locally-advanced tumors [9, 10].

## Pathology and molecular biology

The pathological diagnosis should be made according to the World Health Organization (WHO) classification. The two most frequent subtypes are invasive carcinoma of no special type, which accounts for approximately 70–75%, and lobular carcinoma, representing between 12 and 15% of all breast cancers. The other ≤ 5% are rare histologies, each with its distinct pathologic features and prognosis [11, 12]. Tumor grade and the presence of in situ carcinoma are also relevant features to determine.

Estrogen receptor (ER) and progesterone receptor (PgR) expression should be ascertained by immunohistochemistry (IHC), and reporting of the new category ER Low Positive should be done for those tumors with 1 to 10% positive staining [13]. HER2 status should also be determined as per ASCO-CAP guidelines (I, A) [14]. Proliferation markers such as Ki67 yield additional, useful information. Nevertheless, the high inter-observer variability in the Ki67 determination must be taken into consideration when using it for decision-making [15].

Recent years have witnessed the emergence of new markers in an attempt to substantiate differences in pathogenesis, treatment response, and prognosis:

Tumor-infiltrating lymphocytes (TILs) have proven to predict pathological complete response (pCR) to CT and good prognosis in triple negative and HER2-positive breast cancer [16, 17]. Nevertheless, its use for treatment selection is discouraged at present [18].

In the early, HR positive, HER2-negative setting, various platforms are available (Oncotype^®^, ProsignaTM^®^, Mammaprint^®^, and Endopredict^®^) and can be of value for adjuvant treatment decision in pre- or post-menopausal patients with node-negative disease and in post-menopausal patients with 1–3 positive lymph nodes [19-21].

In HER2-positive disease, the HER2DX 27-gene test has recently emerged as a useful clinical tool [22-25]. The HER2DX test provides two independent scores indicating prognosis when treated with trastuzumab-based CT (HER2DX risk-score), and the probability of achieving a pathological complete response following trastuzumab-based therapy (HER2DX pCR-score). Thus, HER2DX can help to identify suitable candidates for escalation and de-escalation treatment strategies in some clinical situations, even when this tool needs additional validation (II, B).

## Local–regional therapy

### Surgery

Surgical treatment of breast cancer (BC) may consist of tumor excision with surrounding normal breast tissue BCS or mastectomy [26].

Long-term follow-up of randomized clinical trials has revealed similar survival rates for women treated with either BCS or mastectomy (I, A) [27]. Mastectomy is indicated in the following cases: locally advanced tumors, tumor multicentricity, small breast size for tumor volume, inability to achieve negative surgical margins after multiple resections, and contraindications to radiotherapy [28, 29].

Currently, an increasing percentage of women with BC stage II or III receive primary neoadjuvant systemic therapy (NST). In these patients, surgical and radiation treatments are based on the tumor’s initial stage and response to NST. The absence of ink in the tumor comprises a sufficient surgical margin in most cases of BC (I, A) [30].

Patients with BC and germline BRCA1/2 mutations can be considered for BCS, with local control similar to that of non-carriers (I, A). The increased risk of contralateral BC and of new cancers in the ipsilateral breast warrant discussing bilateral mastectomy with the patient (I, A). Nipple-sparing mastectomy is a reasonable approach in these women, provided there is adequate distance from tumor surgical margin [31].

Sentinel lymph node biopsy (SLNB) is standard for patients undergoing BCS with clinically negative axilla assessed by ultrasound imaging (I, A) [32]. Axillary lymph node dissection (ALND) can be omitted after SLNB with one or two positive lymph nodes following mastectomy provided that regional nodal irradiation (RNI), including the axilla, is planned (I, A). ALND may be omitted after SLNB with one or two positive lymph nodes post-BCS for tumors > 5 cm if RNI is planned (III, B). In women undergoing mastectomy with positive sentinel lymph nodes for whom radiation is not planned, complete axillary dissection is recommended (III, B) [30, 33].

Clinically node-positive patients after NST are advised to undergo complete axillary dissection (I, A). SLNB is a post- NST staging procedure for cN0 patients (I, A). In those with a clinically positive axillary node (cN1) who achieve a complete clinical response after NST, axillary dissection can be avoided if ≥ 3 sentinel nodes are identified and all are negative, or when the marked involved node(s) at diagnoses is/are removed, in addition to the sentinel node, and all of them are free of tumor cells [34]. Any residual nodal disease after NST on sentinel node biopsy usually warrant ALND (RT trials are on-going). Patients with cN2 axillary disease should undergo ALND, regardless of response to NST (I, A) [30, 35, 36].

### Adjuvant radiotherapy

After BCS, adjuvant RT is the standard treatment (I, A) [37, 38]. Hypofractionated whole breast irradiation (WBI) represents the preferred approach, with a treatment duration of 3–4 weeks (I, A). Currently, five-fraction WBI regimens have also emerged as standard of care for certain patients based on data from the FAST and FAST-Forward trials (I, A) [39]. Omission of breast irradiation in elderly patients with low risk ER-positive tumors is considered a safe option, although a higher incidence of local recurrence is expected (I, A) [40].

In cases of involvement of ≥ 4 axillary lymph nodes, regional nodal irradiation (RNI) is recommended, as it increases survival in node-positive BC (I, A) [41]. In cases of 1–3 positive lymph nodes, if there are adverse prognostic factors, such as triple negative, HER2, luminal B cancers, and in women with residual disease after NST, experts recommend RNI, regardless of whether mastectomy or BCS have been performed (I, B). Similarly, postmastectomy radiotherapy to the chest wall and regional lymph nodes is recommended in cases of ≥ 4 node-positive nodes (I, A).

## Principles of adjuvant systemic therapy

Breast cancer (BC) is a heterogeneous disease, with different subtypes having a distinct biological, molecular, and clinical outcome. Systemic adjuvant treatment is commonly used in early breast cancer with the intention of lowering the rate of locoregional or systemic relapses and death derived from the disease. Treatment decisions are based on clinical (age, comorbidities) and pathologic factors (tumor size, nodal status, grade, Ki67, HR status, and HER2 status). Multigenic tests provide information beyond standard clinical and pathologic prognostic factors that can aid in making treatment decisions.

## Prognostic gene expression-based assays

Gene expression-based assays, such as OncotypeDx Recurrence Score, Mammaprint, Endopredict, and Prosigna can be used to gain additional prognostic and/or predictive information regarding the benefit of adjuvant CT in early HR-positive and HER2-negative BC (I, A) (Table 2). New data have recently been reported to inform adjuvant ET and CT use on the basis of patient age, menopausal status, and number of axillary nodes involved (II,B) [19, 42-49] (Tables 2 and 3).

**Table 2:** Types of prognostic gene expression-based assays

Platform	Description	Validated	Site	Technology	Risk classification	Prospective randomized study	Evidence
Oncotype	21-gene signature	N0 (pre and postmenopausal) N1(postm)	Central	Microarray	Recurrence Score (RS) Low, Intermediate, High	TAILORx [46] RxPONDER [50]	IA
Mammaprint	70-gene signature Tumoral subtype Blue Print (Luminal, Basal, ERBB2)	N0 N1 (postm)	Central	RT-qPCR	Ultralow, Low, High	MINDACT [45]	IA
Endopredict	11-gene signature	N0 N1 (postm)	Local labs	RT-qPCR	Low, High EPclin	No	IB
Prosigna	50-gene signature tumoral subtype (Luminal A, B, HER2-enriched, Basal-like)	N0 (postm)	Local labs	nCounter (Direct mRNA counting)	Low, intermediate, high (ROR) Intrinsic subtypes (15–19)	OPTIMA (in process)	IB

**Table 3:** SEOM clinical practice guidelines for early breast cancer (2022): summary of recommendations

Recommendation	Category, grade
Diagnosis and initial workup	
Bilateral mammography and ultrasound of breast and regional lymph nodes in patients with suspected breast cancer	I, A
Core needle biopsy (preferably under ultrasound or stereotactic guidance) in patients with suspected breast cancer	I, A
Fine needle aspiration or core biopsy of suspicious lymph nodes	II, A
Immunohistochemical evaluation of estrogen and progesterone receptors together with HER2 expression (following ASCO-CAP guidelines) should be performed in the breast biopsy	I, A
Bilateral breast MRI, with histologic confirmation of additional findings, as part of initial staging in cases of positive axillary nodes; occult primary breast cancer; Paget’s disease of the nipple; lobular carcinoma; multifocal, multicentric lesions, and breast cancer implants	I, B
Bilateral breast MRI is recommended before and after neoadjuvant treatment to define the extent of disease and monitor response to treatment	III, A
Laboratory testing as part of initial staging of patients with confirmed breast cancer	III, C
Additional staging with chest and abdomen CT and bone scan in patients with stage III disease and/or with clinical or laboratory findings suggestive of metastases	III, B
Staging PET/CT can be of use when traditional imaging test are equivocal	III, A
Evaluation of cardiac function in patients requiring anthracyclines and/or trastuzumab	I, A
Surgery	
Consideration of BCS as first surgical option in stages I-II. Mastectomy is indicated in cases of tumor multicentricity, small breast size for tumor volume, inability to achieve negative surgical margins after multiple resections, and contraindications to radiotherapy	I, A
No indication of additional excision in patients with no ink on invasive tumor or DCIS after BCS	I, A
Patients with BC and germline BRCA1/2 mutations can be considered for BCS with similar local control rates. Bilateral mastectomy should be offered as part of an appropriate counseling process in BRCA1/2 mutation carriers	I, A
Sentinel lymph node (SLN) biopsy is standard in patients with clinically negative axillary nodes	I, A
Axillary lymph node dissection should be omitted in patients with stage I–II disease and < 3 positive axillary nodes after SLN biopsy and lumpectomy followed by adjuvant systemic therapy and radiotherapy	I, A
Axillary lymph node dissection may be omitted in patients with stage I–II disease and < 3 three positive axillary nodes after SLN biopsy and mastectomy, provided that adjuvant systemic therapy and regional nodal irradiation including the axilla is indicated	III, B
In patients with cN0 tumors, SLNB is the standard axillary staging procedure after NST	I, A
In patients with cN1 receiving NST, ALND might be avoided in patients with downstaging of axilla to clinically negative if three or more sentinel nodes are identified and all of them are negative, or when the involved node(s) marked at diagnoses is/are removed as well as the sentinel node and all are free of tumor cells	II, B
In patients receiving NST, ALND should be performed in women with any residual disease on sentinel node biopsy	I, A
In patients with cN2-3 tumors receiving NST, ALND should be performed regardless of response to NST	I, A
Adjuvant radiotherapy	
Adjuvant radiation therapy (RT) is the standard treatment after BCS	I, A
Hypofractionated schemes are preferred for external beam whole radiation therapy after BCS	I, A
Breast irradiation may be safely omitted after BCS in elderl low-risk ER-positive tumors assuming a higher rate of local recurrence	I, A
Regional nodal irradiation should be administered in patients with ≥ 4 involved nodes after BCS or mastectomy	I, A
Regional nodal irradiation is recommended in patients with 1–3 involved nodes after BCS or mastectomy in cases with adverse prognostic factors (triple negative, HER2, luminal B cancers)	I, B
Regional nodal irradiation is recommended in patients with residual nodal disease after NST and BCS or mastectomy	I, B
Postmastectomy radiation therapy to the chest wall and regional node irradiation should be administered in patients with ≥ 4 involved nodes	I, A
Decision-making for systemic adjuvant treatment in HR-positive HER2-negative breast cancer	
In breast cancer with HR-positive and HER2 negative, genomic platforms are not recommended in clinically low-risk patients (pT1a-b N0, low grade, ER high) and/or in patients who are not eligible for CT	I, D
In breast cancer with HR-positive and HER2 negative genomic platforms are not recommended in: 1–3 involved nodes coexisting with other high-risk factors and/or premenopausal patients, or with > 3 positive nodes for whom adjuvant CT is indicated	I, D
Oncotype Dx is recommended in premenopausal patients with node negative tumors to predict benefit from adjuvant CT	I, A
Oncotype DX and MammaPrint may be used to guide adjuvant treatment in postmenopausal or > 50 year old patients with node negative disease or 1–3 positive nodes	I, A
EndoPredict may be used to guide adjuvant treatment in postmenopausal or > 50 year old patients with node negative disease or 1–3 positive nodes	II, B
Prosigna may be used in postmenopausal patients with node negative tumors	II, B
Dynamic changes of Ki67 after 2 weeks of preoperative ET in postmenopausal women may be considered as a surrogate prognostic factor	II, B
Adjuvant and neoadjuvant systemic treatment of luminal breast cancer	
Adjuvant therapy should be started before 12 weeks after surgery	I, A
Adjuvant ET should be offered to any patient with HR positive disease (ER or PgR, if either or both are positive defined as ER and/or PR > 1/10%), regardless of other prognostic factors	I, A
Recommendations for endocrine therapy in premenopausal patients	
Adjuvant ET with tamoxifen for five years is recommended as a standard treatment for low-risk premenopausal women with HR positive breast cancer	I, A
Extended adjuvant ET with tamoxifen for up to 10 years should be considered in high-risk patients who remain premenopausal during the entire adjuvant period	I, B
Ovarian function suppression plus ET (preferentially with an AI) should be considered in high-risk premenopausal patients who recover ovarian function in the first 12–18 months after CT	I, A
In patients treated with ovarian function suppression, regular monitoring of estrogen levels should be performed during the first year, especially in younger patients in whom OFS is achieved with LHRH analogues	I, A
In premenopausal patients becoming postmenopausal during the first 2–5 years of tamoxifen, a switch to aromatase inhibitor should be considered after evaluating the risk of late recurrence	II, A
Recommendations for endocrine therapy in postmenopausal patients	
For postmenopausal women, both non-steroidal and steroidal AI are superior to tamoxifen	I, A
Adjuvant ET for postmenopausal patients may consist of any of the following alternatives, after considering risk factors and individual preferences: Upfront AI AI after 2–3 years of tamoxifen AI after 5 years of tamoxifen (letrozole and anastrozole) as extended adjuvant therapy, especially in intermediate- to high-risk (node positive) patients	I, A
Extended adjuvant therapy (optimal duration: 7.5–8 years) should be discussed with nearly all patients, except those with a very low risk of relapse	I, A
Extended adjuvant therapy with AI for more than 8 years offers minimal benefit	I, C
In high-risk postmenopausal patients who decline or do not tolerate AI, 10 years of tamoxifen should be considered	I, A
General recommendations for adjuvant treatment	
Adjuvant bisphosphonates are recommended in women with low-estrogen status and/or treatment-related bone loss	I, A
Adjuvant abemaciclib for 2 years in combination with adjuvant ET should be considered in high-risk patients (defined as tumors with ≥ 4 positive nodes or 1–3 nodes and either tumor size > 5 cm, histologic grade 3, or Ki-67 > 20%)	I, A
Adjuvant olaparib for 1 year in combination with adjuvant ET should be considered in patients with germline pathogenic BRCA mutations, treated with adjuvant or NAC and with high-risk tumors (defined as tumors with ≥ 4 positive nodes in the adjuvant setting or as a CPS + EG score of ≥ 3 without pCR in the neoadjuvant setting)	I, A
Recommendations for adjuvant chemotherapy	
Adjuvant CT for HR + HER2-negative breast cancer is recommended for tumors defined as high-risk tumors defined by either clinical or genomic characteristics: T2-4 and/or axillary node involvement N2-3, extensive LVI, high Ki67, low ER expression, younger age or premenopausal status, and intermediate- to high-risk genomic score	I, A
Sequential anthracycline/taxane-based regimen is the standard for most patients	I, A
CT should be administered for 12–24 weeks (4–8 cycles)	I, A
AC or EC are the standard anthracycline-based regimens, which should not include 5-FU	I, A
The use of dose-dense schedules (with granulocyte colony-stimulating factor support) should be considered in high-risk tumors	I, A
In selected lower-risk patients, 4 cycles of anthracycline- or taxane-based CT or CMF may be used	II, B
Non-anthracycline regimens may be used in patients at risk for cardiac complications	I, A
Recommendations for male patients with breast cancer	
In male patients with HR + HER2-negative breast cancer, tamoxifen is the standard treatment	III, A
In male patients with HR + HER2-negative breast cancer and a strong contraindication for tamoxifen, a combination of an AI plus a luteinizing hormone-releasing hormone agonist may be considered	III, B
CT indications and regimens should follow the same recommendations as those for breast cancer in female patients	III, A
Recommendations for neoadjuvant therapy	
CT drugs and drug regimens used in the preoperative setting should be selected according to rules identical to those in the postoperative setting	I, A
A sequential regimen of anthracyclines and taxanes is recommended in those patients in whom NAC is indicated for HR-positive and HER2 negative breast cancer	I, B
NET alone may be offered to those postmenopausal patients with strongly HR-positive tumors (RE > 60% or RE 40–60% and PR > 10%)	I, A
NET in postmenopausal patients should include an aromatase inhibitor during at least 6–8 months or until maximum response	II, B
NET with AI plus ovarian suppression might be considered in highly selected premenopausal patients with luminal A tumors with no indication for CT and who are not candidates for optimal surgery	II, C
Neoadjuvant and adjuvant systemic treatment of HER2 breast cancer	
Patients with HER2-positive tumors > 2 cm tumor size and/or node-positive disease should be treated with NST including dual HER2 blockade with trastuzumab and pertuzumab and CT with sequential taxanes/anthracyclines or taxane/carboplatin combinations	I, A
Selection of neoadjuvant regimens without anthracyclines may be used if seeking to avoid cardiotoxicity	II, B
HER2Dx may be used to provide estimates of the likelihood of achieving pCR and of the risk of recurrence	II, B
Addition of standard 12-month adjuvant trastuzumab to CT is recommended for HER2 positive breast cancer both in node-positive and in node-negative tumors with a tumor size > 1 cm	I, A
Addition of adjuvant trastuzumab to CT may be considered in cases of node-negative HER2 positive breast cancer with tumor size of 0.5–1.0 cm	II, B
For adjuvant CT of HER2 positive breast cancer, 4 cycles of AC or EC followed by 3 months of paclitaxel (P) or docetaxel (D) or both in combination with trastuzumab (AC/EC P/D + H) or docetaxel, carboplatin and trastuzumab (TCH) are the preferred regimens	I, A
In node-negative, stage I, HER2-positive tumors single-agent weekly paclitaxel and trastuzumab for 12 weeks followed by single-agent trastuzumab (to complete one year) should be considered	II, B
Adjuvant dual HER2 blockade with trastuzumab and pertuzumab for 18 cycles may be considered in patients with node-positive, HER2-positive breast cancer. In clinically node-positive patients that have received neoadjuvant treatment, up to 18 cycles of pertuzumab may be continued after surgery	II, B
Extended adjuvant treatment with neratinib after one year of trastuzumab may be considered in patients with node positive and HR-positive HER2-positive breast cancer	I, B
In patients with pCR after standard NST, adjuvant therapy with trastuzumab should be administered until one full year of total anti-HER2 therapy has been completed	I, A
Adjuvant T-DM1 for 14 cycles, instead of trastuzumab, should be considered in patients with HER2-positive breast cancer and residual disease after standard NST	I, A
In patients with HER2-positive and HR + breast cancer, adjuvant ET should be administered following the same principles as in HER2-negative HR + disease	I, A
Adjuvant and neoadjuvant systemic treatment for triple negative breast cancer	
When upfront surgery followed by adjuvant CT is the preferred option for triple negative breast cancer, the regimen should include an anthracycline and a taxane, although a taxane-cyclophosphamide combination or taxane monotherapy might be an alternative in patients at high risk for cardiac toxicity	I, B
Adjuvant CT may be considered for 0.6–1 cm tumors after discussing potential risks and benefits with the patient	III, B
NAC for triple negative breast cancer should include anthracyclines and taxanes, preferably with dose-dense sequential regimens	I, A
Carboplatin improves the pCR rate and event-free survival and may be considered as part of NAC for triple negative breast cancer patients	I, B
Addition of neoadjuvant pembrolizumab to NAC should be considered in the neoadjuvant setting for triple negative breast cancer irrespective of PD-L1 expression. Adjuvant pembrolizumab might be administered as adjuvant treatment	I, B
Adjuvant capecitabine for 6–8 cycles should be considered in high-risk, triple negative breast cancer with residual invasive disease at surgery following standard NAC	I, B
Adjuvant olaparib for 1 year should be considered in individuals with germline BRCA1/2 mutations and high-risk triple negative breast cancer with residual invasive disease at surgery following standard NAC	I, B
Follow-up of early breast cancer	
Healthy lifestyles, especially an active lifestyle, are recommended to prevent tumor recurrence and to improve quality of life	II, B
For early breast cancer, regular follow-up visits are recommended every 3–6 months during the first 2 years, every 6 months from years 3–5, and annually thereafter	III, A
Annual ipsilateral (after BCS) and/or a contralateral mammography is recommended for follow-up of early breast cancer	II, A
MRI of the breast may be considered for follow-up of young patients with dense breast tissue or with genetic or familial predisposition	II, B
Ultrasound or contrast-enhanced mammography may be considered as an additional study under the indication of a radiologist in doubtful cases or when there is a contraindication to MRI	III, B
There is no demonstrated survival benefit of including tumor markers or imaging tests (other than breast imaging) in the follow-up of asymptomatic patients	I, D

Recommendations:

- Genomic platforms are not recommended for the following: clinically low-risk tumors (pT1a, b, node negative, low grade, ER-high) and/or patients with health conditions who are not candidates for CT (I, D).
- Genomic platforms are not recommended for the following: 1–3 involved nodes coexisting with other high-risk factors and/or premenopausal patients, or patients with > 3 positive nodes for whom adjuvant CT is indicated (I, A).
- Oncotype Dx is recommended in premenopausal patients with node negative tumors (IA). Based on TailorX results, CT has some benefit for distant recurrence if the RS is 16–25 (II, B).
- Oncotype DX, MammaPrint, and EndoPredict can be useful to guide adjuvant treatment in postmenopausal or > 50 year patients with node negative or 1–3 positive nodes (I, A; I, A; II, B) [50].
- Prosigna may be used in postmenopausal patients with node-negative tumors (II, B) [51].
- Dynamic changes in Ki67 after 2 weeks of perioperative ET in postmenopausal women can be considered a surrogate prognostic factor based on the POETIC trial (II, B) [52].

## Systemic treatment for early-stage luminal-type breast cancer

### Adjuvant endocrine therapy for early-stage breast cancer

**Fig. 1:**
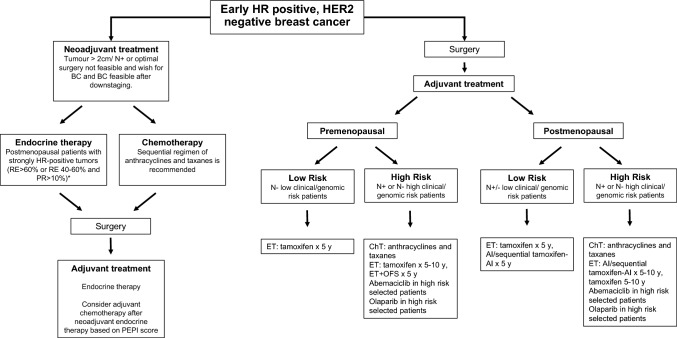
HR positive/HER2-negative early breast cancer algorithm. *AI:* aromatase inhibitors; *BCS:* breast conservation; *CT:* chemotherapy; *ET:* endocrine therapy; *N:* axillar node; *OFS*: ovarian function suppression; *y:* years; +:  positive; -: negative. *Consider in premenopausal patients with luminal A-like tumors and no indication for chemotherapy, who are not candidates for optimal surgery, *OFS* plus an aromatase inhibitor can be considered

There is robust evidence that ET improves survival of early-stage luminal breast cancer (BC). Adjuvant ET should be offered to all ER + patients regardless of age, menopausal status, CT exposure, hormone expression level (ER or PgR) (if any or both are positive defined as ER and/or PgR > 1/10%), and/or Her2 status (I,A) [53]. There are several ET options. The individual choice should be adjusted to menopause status, comorbidity, and risk of recurrence. Adjuvant systemic therapy is best started without undue delays, as data reveal an important decrease in efficacy when it is administered > 12 weeks after surgery (I, A) [54] (Fig. 1).

Recommendations for premenopausal patients:

- Tamoxifen for 5 years is the most widely established adjuvant ET for low-risk premenopausal patients (I, A) [55]. Consider tamoxifen until 10 years in high-risk tumors in the presence of ovarian function (at the expense of greater toxicity) (I, B) [56, 57].
- In high-risk premenopausal patients who recover menses or ovarian function after CT (in the first 12–18 months), the addition of ovarian function suppression + ET should be offered (I, A), being the most effective combination ovarian function suppression (OFS) with AI [58, 59].
- In patients treated with AI + chemical OFS, clinicians should control estrogen levels biochemically at regular intervals (I,A), mainly in younger patients during the first year of treatment. LHRH analogues should be administered in the monthly schedule for these patients, for a total duration of 2. 5 to 5 years (I, A)
- In patients becoming postmenopausal during the first 2–5 years of tamoxifen, a switch to aromatase inhibitor should be considered, depending on risk of late recurrence (II,A).Total duration of adjuvant ET should be 7.5 to 8 years if intermediate or high clinical risk of relapse (I, A)

Recommendations for postmenopausal patients:

- For postmenopausal women, AIs (both non-steroidal and steroidal) are superior to tamoxifen (I, A), although tolerance and toxicity profiles should be individualized.
- AIs can be used upfront (non-steroidal AI and exemestane), after 2–3 years of tamoxifen (non-steroidal AI and exemestane), or as extended adjuvant therapy, after 5 years of tamoxifen (letrozole and anastrozole) (I, A), especially in intermediate- to high-risk patients [60-65].
- Extended adjuvant therapy should be discussed with all patients. Except those with a very low risk of relapse (I, A), the optimal duration of ET should be of 7.5 to 8 years. There is only a minimal benefit from the use of AIs for more than 8 years (I, C) [66]. The predictive benefit of Breast Cancer Index for extended ET has been demonstrated in various cohorts that include patients with 0–3 involved lymph nodes [67-69].
- The option of 10 years of tamoxifen in postmenopausal patients could be considered in high-risk patients who decline or have a contraindication to AIs (I, A).
- Biphosphonates are recommended in women with low-estrogen status and also in those with treatment-related bone loss (I, A) [70].
- For high-risk patients, defined as tumors with ≥ 4 positive nodes, or 1–3 nodes and either tumor size > 5 cm, histologic grade 3, or Ki-67 > 20%, abemaciclib for 2 years in combination with ET is indicated for adjuvant treatment based on the MonarchE trial (I, A) [71]
- In the Olympia trial, 1 year adjuvant olaparib demonstrated improved OS and DFS in patients with HER2-negative breast cancer with *BRCA1* or *BRCA2* germline pathogenic mutations, and high-risk clinic-pathological features (at least four pathologically-confirmed positive lymph nodes in the ER + population).Triple negative or ER + patients treated with NAC who don´t achieve a pCR ( CPS + EG score of ≥ 3 in the ER + group) are also candidates for this treatment (I, A) [72]. Despite this evidence, olaparib is still awaiting financial approval from the health authorities in Spain.

### Adjuvant chemotherapy in hormone receptor-positive early BC

The use of CT as adjuvant treatment for ER + Her2-negative disease is recommended for high-risk tumors defined by either clinical or genomic profiling characteristics (I, A), considering: T2 to T4 tumors and/or axillary N2-3 involvement; high Ki67; low ER expression; younger age or premenopausal status, and intermediate- to high-risk genomic score.

Recommendations:

- CT should be administered for 12–24 weeks (4–8 cycles) (I, A).
- Sequential anthracycline/taxane-based regimen is the standard for most patients (I, A). Anthracycline-based regimens should not include 5-FU (EC or AC is standard) (I, A) [73, 74].
- In selected lower-risk patients, 4 cycles of taxane-based CT or CMF may be used (II, B) [75, 76].
- Non-anthracycline regimens may be used in patients at risk for cardiac complications (I, A) [76].
- The use of dose-dense schedules (with granulocyte colony-stimulating factor support) should be considered in high-risk tumors (I, A) [77, 78].

In male patients, tamoxifen is the standard adjuvant systemic therapy (III, A); AIs should not be used alone in this setting. If a strong contraindication exists for the use of tamoxifen, a combination of an AI plus a luteinizing hormone-releasing hormone agonist may be considered (III, B). CT indications and regimens should follow the same recommendations as those for breast cancer in female patients (discus with patients higher toxicity and compliance) (III, A) [79-81].

### Neoadjuvant treatment in hormone receptor-positive early BC

Neoadjuvant treatment is recommended in locally advanced tumors and in those situations where decrease the extent of surgery is needed (I, A). The timing of treatment (pre- versus postoperative) has no effect on long-term outcomes, except a possible small increase in locoregional recurrences, but without impact on survival (II, A) [82].

Recommendations:

- NET alone may be offered to those postmenopausal patients with strongly HR-positive tumors (RE > 60% or RE 40–60% and PgR > 10%) (I, A). AIs are more effective than tamoxifen in decreasing Ki67 levels, tumor size and facilitating less extensive surgery (I, A) [83, 84].
- The preferred ET option for postmenopausal patients is an aromatase inhibitor during at least 6–8 months or until maximum response (II, B).
- NET is not routinely recommended in premenopausal patients, outside clinical trials. However, in highly selected patients with luminal A-like tumors and no indication for CT, who are not candidates for optimal surgery, OFS plus an aromatase inhibitor can be considered (II, C) [85].
- Some phase II trials and one meta-analysis showed similar response rates comparing NET and CT, but a significantly lower toxicity with NET (II, B) [86].
- The efficacy evaluation of NET has been performed according to surrogate parameters such as the decrease of the Ki67 levels during the first cycle of NET, or the preoperative endocrine prognostic index (PEPI) score after surgery (II, B) [87].
- Different genetic signatures have been evaluated in core needle biopsy before neoadjuvant therapy, as good predictors of response to neoadjuvant therapy, especially PAM50 ROR score, although this approach is currently considered experimental (II, C) [88].
- CT drugs and drug regimens used in the preoperative setting should be selected according to rules identical to those in the postoperative setting (I, A). A sequential regimen of anthracyclines and taxanes is recommended for the vast majority of patients (I, B).

## Systemic treatment for HER2-positive early breast cancer

### Neoadjuvant treatment for HER2-positive disease

Neoadjuvant treatment in HER2 positive breast cancer provides a useful information on pathological response that is a surrogate marker of DFS and potentially overall survival [89] (OS) and also, opens the window to the knowledge of residual disease and to tailor adjuvant strategies after surgery.

**Fig. 2:**
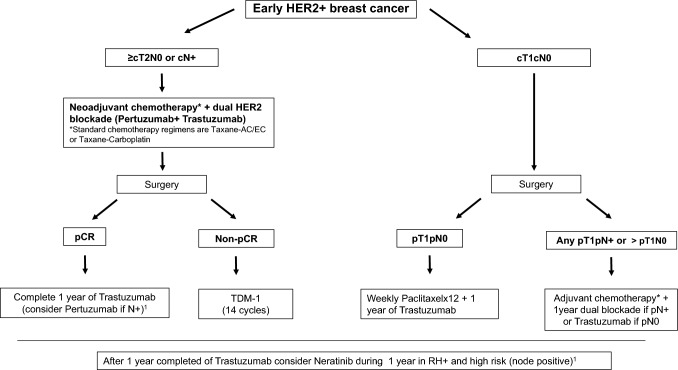
HER2-positive early breast cancer algorithm. *CT:* chemotherapy. *pCR:* pathologic complete response. (1) This treatment is still awaiting financial approval from the health authorities in Spain

- Patients with ≥ cT2 tumors or cN + should be treated with standard CT (Taxane-AC/EC or Taxane-Carboplatin) plus dual HER2 blockade, Pertuzumab (P) + Trastuzumab (T) [90]. This dual HER2 blockade efficacy has been endorsed by the latest analysis of CLEOPATRA study confirming OS benefit in the advanced setting [91] (I, A) (Fig. 2)
- Established NAC regimens are either an anthracycline-taxane sequence plus P + T or docetaxel-carboplatin plus dual HER2 blockade, for a minimum of 9 weeks of taxane + antiHER2 therapy.
- Recently, the TRAIN2 study suggested that an anthracycline combination does not add efficacy neither regarding pCR nor patient outcome to a sequential taxane-platinum containing regimen with dual antibody blockade [92]. The evidence for anthracycline-free CT in HER2 + early BC is reinforced in the neoadjuvant setting in TRYPHAENA trial and in the adjuvant setting in BCIRG 006. The incidence of significant declines in the left ventricular ejection fraction (LVEF) is lower without anthracycline containing regimen so this could be the chosen CT backbone if desire to avoid cardiotoxicity [93, 94] (II, B).
- HER2DX risk score and pCR score has recently been developed and validated (both based on a 27-gene expression plus clinical feature-based classifier) and will provide accurate estimates of the risk of recurrence, and the likelihood to achieve a pCR, in early-stage HER2-positive breast cancer patients (II, B) [22-25].

### Adjuvant treatment for HER2-positive disease

- The administration of trastuzumab associated with adjuvant CT treatment demonstrated reduction in risk of relapse of 50% and also on mortality, regardless of tumor size, age, nodal and HR status [94-96]. The optimal duration of trastuzumab treatment has been established as 12 months (I, A) [97, 98]. It may be safely combined with either radiotherapy or ET. Adjuvant trastuzumab is recommended in all tumors with a tumor size > 1 cm regardless of nodal status (I, A). Adjuvant trastuzumab might be considered in node-negative tumor size 0.5-1 cm tumors, specially in ER- disease, although no level I evidence exists (II, B).
- Thus, 4 cycles of AC or EC followed by 3 months of paclitaxel (P) or docetaxel (D or T) both in combination with trastuzumab (AC/EC → P/D + H), or docetaxel, carboplatin, and trastuzumab (TCH) are preferred regimens (I, A).
- In stage I, treatment with paclitaxel for 12 weeks associated with trastuzumab should be considered based on the results of the phase II APT trial (II, B) [99].
- In the Aphinity trial [100, 101], the addition of pertuzumab demonstrated a modest but significant benefit in invasive DFS (iDFS) in the node-positive cohort, regardless of HR status. No statistically significant difference in OS was found. Based on this trial, the EMA approved the use of 18 cycles of dual T + P treatment in the high-risk node-positive population, regardless of whether it was initiated in the adjuvant or the neoadjuvant setting (II, B). Despite this evidence, pertuzumab is still awaiting financial approval from health authorities in Spain.
- The addition of 1 year of adjuvant neratinib improved iDFS in patients with HER2-positive breast cancer after 1 year of trastuzumab, as demonstrated in the phase III EXTENET trial [102]. The benefit was greater in patients with HR-positive and node-positive disease, at the expense of increased toxicity (diarrhea) (I, B). Neratinib has been approved by EMA, which restricted its use to HR + disease. The authorities in Spain also restrict its use to subjects who had not completed one year of trastuzumab for any reason.
- In those patients who receive neoadjuvant treatment (minimun 6 cycles with at least 9 weeks of taxane + trastuzumab regimen) and who do not achieve pCR, 14 cycles of adjuvant T-DM1 substantially improve outcomes compared with adjuvant trastuzumab (KATHERINE trial) with a substantial difference in 3-year iDFS (88.3% vs 77%). This benefit is seen independently of adjuvant ET, radiotherapy or HER2 status in the residual disease [103] (I, A)
- In patients with HER2-positive HR + BC, adjuvant ET should be administered following the same principles as in HER2-negative disease.

In patients with pCR, adjuvant anti-HER2 therapy with trastuzumab for a full year of total anti-HER2 therapy should be maintained (I, A). Patients with node-positive disease at diagnosis may receive pertuzumab added to trastuzumab if we extrapolate the results of the adjuvant APHINITY trial [100] (II, B).

## Systemic treatment for triple negative breast cancer

### Adjuvant treatment for triple‐negative disease

Triple negative breast cancer (TNBC) is a heterogeneous disease that accounts for approximately 15–20% of all breast cancers [104]. It tends to comprise high-grade tumors with a high proliferation index and a particular trend to metastasize early to different organs such as liver, lung, and central nervous system (CNS) [105].

**Fig. 3:**
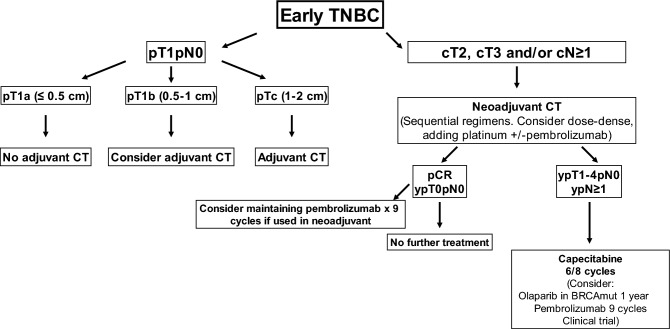
Triple negative early breast cancer algorithm. *CT:* chemotherapy. *pCR:* pathologic complete response

While historically surgery and adjuvant CT have been considered the cornerstone of early TNBC treatment, NST has emerged as the preferred option not just in locally advanced tumors but in smaller tumors as well. Since systemic therapy should be considered in all stage I tumors (except in those ≤ 5 mm, with an excellent prognosis without CT) [106], administration of systemic neoadjuvant therapy could yield locoregional benefits and offer response information (Fig. 3).

Nevertheless, when upfront surgery and adjuvant CT is the preferred option, the regimen should include an anthracycline and a taxane (I, B), although a taxane/cyclophosphamide or taxane/platinum combination can represent a good alternative in patients with potential cardiac toxicity (I, B). CT should be discussed with patients with pT1b N0 (6–10 mm) tumors, weighing potential risks and benefits in this good prognosis group [106] (III, B).

### Neoadjuvant treatment for triple‐negative disease

NST with CT or NAC is the preferred approach in locally advanced (stage II-III) TNBC, leading classically to pCR rates of 30–40%. Patients who achieve a pCR have an excellent prognosis. Residual disease after NAC is a recognized biomarker associated with an increased recurrence risk that can be very useful when selecting patients for post-neoadjuvant escalating therapies.

The combination of anthracyclines and taxanes is the treatment of choice (I, A), preferably with dose-dense sequential regimens [77]. The addition of platinum compounds to standard sequential anthracycline and taxane regimens has largely remained a controversial issue. Overall, based on the results of a meta-analysis that included nine randomized controlled trials investigating platinum-based versus platinum-free NAC in TNBC, an increase in pCR from 37.0 to 52.1% was observed with platinum-based regimens [107]. While none of these studies were designed to determine benefit in DFS or OS, the recently published long-term results of the Brightness trial have confirmed that improved pCR rates with the addition of carboplatin were associated with long-term EFS benefit, including in BRCA1/2 carriers [108] (I, B).

The recent incorporation of immunotherapy to NAC regimens for TNBC has changed the “chemotherapy-only” neoadjuvant approach in these patients. In the phase III KEYNOTE-522 trial, the addition of pembrolizumab demonstrated a significantly higher pCR rate (64.8% vs 51.2%) and 18-month EFS (91.3% vs. 85.4%) [109] (I, B). Although the PD-L1-positive subgroup had higher overall pCR rates, benefit was observed regardless of PD-L1 expression. The smaller phase III trial, IMpassion031, that evaluated atezolizumab as the immunotherapy agent, has confirmed a similar benefit in pCR rates [110] but without any significan impact in EFS.

As stated above, patients with residual disease after NAC have a significant risk of disease recurrence, particularly in the first 2–3 years following diagnosis. In the postneoadjuvant setting, the administration of 6–8 cycles of capecitabine represents a good therapeutic option, based on the results of the CREATE-X trial (9) and a recently published meta-analysis [111] (I, B). In the Olympia trial BRCA1/2 mutation carriers with TNBC treated with NAC who did not achieve a pCR derived a significant increase in DFS with 1 year of adjuvant olaparib (I, B) [112]. In patients treated with neoadjuvant pembrolizumab according to the Keynote-522 regimen, pembrolizumab should be administered as adjuvant treatment, although its value in patients obtaining near-pathological complete response (RCB 0 or I) is unclear (I, B) [109].

## Follow-up, long-term implications, and survivorship

Breast cancer follow-up should focus on detecting disease relapse or second primary neoplasms. Although there is no universal sequence or protocol for the follow-up of these patients, taking into account both patient needs and follow-up costs, regular visits are recommended every 3–6 months in the first 2 years, every 6 months after 3–5 years, and annually thereafter (III, A). As part of monitoring, each visit should include a thorough anamnesis, record of symptoms, and a physical examination. Annual ipsilateral (after BCS) and/or contralateral mammography (after mastectomy) is recommended (II, A). A magnetic resonance imaging of the breast may be indicated for young patients, especially in cases of dense breast tissue and genetic or familial predisposition [113] (II, B). Ultrasound may be considered only as an additional study under the indication of a radiologist in doubtful cases or when there is a contraindication for MRI; contrast-enhanced mammography might also be indicated in these cases (III, B) [114]. Routine imaging of reconstructed breast is not indicated.

There are no data to indicate that either laboratory or imaging tests or any tumor markers, such as cancer antigen 15–3 (CA15-3) or carcinoembryonic antigen (CEA), result in a survival benefit (I, D). In symptomatic patients or in the case of abnormal findings on examination, appropriate and specific tests should be performed immediately (III, A) [114, 115].

It is also important to evaluate and manage the toxicities of the treatment received, both physical and psychosocial, in the short and long term. Patients on tamoxifen treatment will require age-appropriate gynecological screening. Those on an aromatase inhibitor or who experience ovarian failure secondary to treatment should undergo monitoring of bone health with a bone mineral density determination at baseline and periodically thereafter [115]. And finally, healthy lifestyle habits, such as an active lifestyle (II, B), a healthy diet, limited alcohol consumption, and achieving and maintaining an ideal body weight (20–25 BMI) can lead to optimal breast cancer outcomes and improved quality of life [114, 115].

## Data Availability

All data supporting the findings of this study are available within the paper.

## Abbreviations

AI: Aromatase inhibitor
ALND: Axillary lymph node dissection
BC: Breast cancer
BCS: Breast conserving surgery
CT: Chemotherapy
DFS: Disease-free survival
EFS: Event-free survival
ER: Estrogen receptor
ET: Endocrine therapy
GEICAM: Grupo Español de Investigación en Cáncer de Mama (Spanish Group for Breast Cancer Research)
HR: Hormone receptor
iDFS: Invasive disease-free survival
MRI: Magnetic resonance imaging
NAC: Neoadjuvant chemotherapy
NET: Neoadjuvant endocrine therapy
NST: Neoadjuvant systemic therapy
OFS: Ovarian function suppression
OS: Overall survival
PgR: Progesterone receptor
pCR: Pathologic complete response
SLNB: Sentinel lymph node biopsy
SOLTI: Grupo español de estudio, tratamiento y otras estrategias experimentales en tumores sólidos (Spanish group of study, treatment and other experimental strategies in solid tumors)
SEOM: Sociedad Española de Oncología Médica (Spanish Society of Medical Oncology)
TNBC: Triple negative breast cancer
WBI: Whole breast irradiation
