# Correction: SEOM-GEICAM-SOLTI clinical guidelines for early-stage breast cancer (2022)

**DOI:** 10.1007/s12094-023-03334-y

**Published:** 2023-10-30

**Authors:** Francisco Ayala de la Peña, Silvia Antolín Novoa, Joaquín Gavilá Gregori, Lucía González Cortijo, Fernando Henao Carrasco, María Teresa Martínez Martínez, Cristina Morales Estévez, Agostina Stradella, María Jesús Vidal Losada, Eva Ciruelos

**Affiliations:** 1 Department of Medical Oncology, Hospital G. Universitario Morales Meseguer, University of Murcia, Av. Marqués de los Vélez, s/n, 30008 Murcia, Spain; 2 Department of Medical Oncology, Complexo Hospitalario Universitario, A Coruña (CHUAC), Coruña, Spain; 3 Fundación Instituto Valenciano de Oncología (IVO), Valencia, Spain; 4 Medical Oncology Department, Hospital Universitario Quirónsalud, Madrid, Spain; 5 Hospital Universitario Virgen Macarena, Seville, Spain; 6 Medical Oncology Department, INCLIVA Biomedical Research Institute, Hospital Clínico of Valencia, University of Valencia, 46010 Valencia, Spain; 7 Hospital Universitario Reina Sofía, Córdoba, Spain; 8 Medical Oncology Department, Institut Català d’Oncologia. L’Hospitalet,, Barcelona, Spain; 9 Hospital Clínic, Barcelona, Spain; 10 Medical Oncology Department, Breast Cancer Unit, University Hospital 12 de Octubre, Madrid, Spain and HM Hospitales, Madrid, Spain

**Correction: Clinical and Translational Oncology (2023) 25:2647–2664** 10.1007/s12094-023-03215-4

In Fig. 3 of this article, the second level of options appears “pT2, pT3 and/or pN ≥ 1” but it should have been “cT2, cT3 and/or cN ≥ 1”. In the last text box, the duration of Olaparib in BRCAmut should be 1 year instead of 2 years. The corrected figure is given below.

**Fig. 3:**
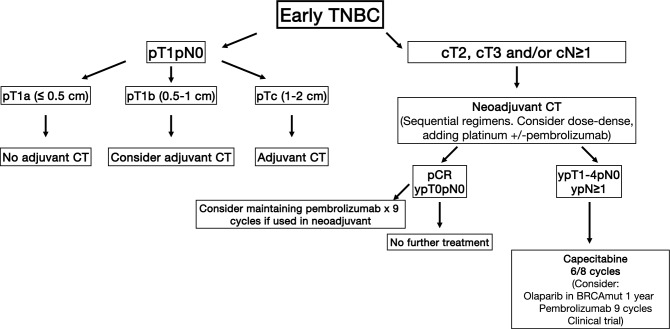
Triple negative early breast cancer algorithm. *CT:* chemotherapy. *pCR:* pathologic complete response

The original article has been corrected.

